# BrainOS: A Novel Artificial Brain-Alike Automatic Machine Learning Framework

**DOI:** 10.3389/fncom.2020.00016

**Published:** 2020-03-03

**Authors:** Newton Howard, Naima Chouikhi, Ahsan Adeel, Katelyn Dial, Adam Howard, Amir Hussain

**Affiliations:** ^1^Etats-Unis, Department of Neurosurgery, Nuffield Department of Surgical Sciences, John Radcliffe Hospital, Oxford, United Kingdom; ^2^REGIM-Lab: REsearch Groups in Intelligent Machines, National Engineering School of Sfax (ENIS), University of Sfax, Sfax, Tunisia; ^3^School of Engineering, Computing and Mathematics, University of Plymouth, Plymouth, United Kingdom; ^4^School of Mathematics and Computer Science, University of Wolverhampton, Wolverhampton, United Kingdom; ^5^Howard Brain Sciences Foundation, Providence, RI, United States; ^6^School of Computing, Edinburgh Napier University, Edinburgh, United Kingdom

**Keywords:** human brain, artificial intelligence, architecture design, hyperparameters, automatic machine learning, BrainOS

## Abstract

Human intelligence is constituted by a multitude of cognitive functions activated either directly or indirectly by external stimuli of various kinds. Computational approaches to the cognitive sciences and to neuroscience are partly premised on the idea that computational simulations of such cognitive functions and brain operations suspected to correspond to them can help to further uncover knowledge about those functions and operations, specifically, how they might work together. These approaches are also partly premised on the idea that empirical neuroscience research, whether following on from such a simulation (as indeed simulation and empirical research are complementary) or otherwise, could help us build better artificially intelligent systems. This is based on the assumption that principles by which the brain seemingly operate, to the extent that it can be understood as computational, should at least be tested as principles for the operation of artificial systems. This paper explores some of the principles of the brain that seem to be responsible for its autonomous, problem-adaptive nature. The brain operating system (BrainOS) explicated here is an introduction to ongoing work aiming to create a robust, integrated model, combining the connectionist paradigm underlying neural networks and the symbolic paradigm underlying much else of AI. BrainOS is an automatic approach that selects the most appropriate model based on the (a) input at hand, (b) prior experience (a history of results of prior problem solving attempts), and (c) world knowledge (represented in the symbolic way and used as a means to explain its approach). It is able to accept diverse and mixed input data types, process histories and objectives, extract knowledge and infer a situational context. BrainOS is designed to be efficient through its ability to not only choose the most suitable learning model but to effectively calibrate it based on the task at hand.

## 1. Introduction

As humans are constantly surrounded by data, their survival depends on their capability to understand and evaluate their observations of the external environment. They formulate and extract knowledge from received information by transforming the data into specific patterns and models. To this end, a number of biological processes and aspects of the brain are involved (Hernandez et al., 2010). Once established, brain agents create and refer to these models with each observation.

Both researchers and theorists specializing in neuroscience agree that these brain agents support the task of analyzing external data, processing them and making decisions using fundamental units of thought. Howard and Hussain (2018) describe this process of the fundamental code unit as cognitive minimums of thought where n to N information exchange is expressed in an assembly-like language at the neuronal cellular level. The Fundamental Code Unit addresses the question of whether input signals feed to the brain in their analogical form or if they are transformed beforehand. Bierdman's theory of components recognition and Yin's review of theories of geometry of perception supports the FCU model where an infinite combination of patterns are created from a fixed number of components (Yin, 2008). The conclusions regarding brain processes derived from the field of neuroscience are applied in parallel to the field of artificial intelligence (AI) (Wang et al., 2016). The finest example of this is Machine Learning (ML), which is inspired by the brain's methods of processing external signals (input data) (Wang et al., 2016). ML can mimic human brain behavior (Louridas and Ebert, 2016) by providing a set of appropriate and intelligent techniques to perform data analysis (Howard and Lieberman, 2014). ML automates data manipulation by extracting sophisticated analytical models. Within this branch of AI, systems are capable of learning from data and distributions, distinguishing patterns and making autonomous decisions, which considerably decreases the need for human intervention.

The appeal of ML is considerably rising due to factors, such as the growing demands of data mining tools (Bredeche et al., 2006). Indeed, in a world replete with data, intelligent computation is gainful in terms of expense and performance (Wang and Yan, 2015). Automated data handling has yielded valuable systems able to solve increasingly complex problems and provide more accurate outcomes.

The three big challenges that ML still face are (1) that it requires a great deal of training data and is domain-dependent, (2) it can produce inconsistent results for different types of training or parameter tweaking, and (3) it produces results that may be difficult to interpret when such black-box algorithms are used. Here, we propose a novel automatic approach to address such shortcomings in a multidisciplinary approach that aims to bridge the gap between statistical Natural Language Processing (NLP) (Cambria et al., 2014) and the many other disciplines necessary for understanding human language, such as linguistics, common sense reasoning and computing. Our proposed approach, “Brain OS” is an intelligent adaptive system that combines input data types, processes history and objectives, researches knowledge and situational context to determine what is the most appropriate mathematical model, chooses the most appropriate computing infrastructure on which to perform learning, and proposes the best solution for a given problem. BrainOS has the capability to capture data on different input channels, perform data enhancement, use existing AI models, create others and fine-tune, validate and combine models to create more powerful collection of models. To guarantee efficient processing, BrainOS can automatically calibrate the most suitable mathematical model and choose the most appropriate computing learning tool based on the task to handle. Thus, it arrives at “optimal” or pre-optimal solutions. BrainOS leverages both symbolic and sub-symbolic methods as it uses models, such as semantic networks and conceptual dependency representations to encode meaning but it also uses deep neural networks and multiple kernel learning to infer syntactic patterns from data. The architecture of BrainOS uses concepts from the critic-selector model of mind and from brain pathology treatment approaches.

Herein, a thorough evaluation of the state of the art of Automatic ML is discussed, and specifically the proposed automatic BrainOS is presented in detail. The advantages of BrainOS over state of the art models are enumerated, and an empirical study is presented in order to validate the proposed framework.

## 2. State-of-the-Art: Automatic ML Frameworks

ML has several models, which apply one or more techniques to one or more applications. ML models include support vector machine (SVM) (Mountrakis et al., 2011), bayesian networks (BNs) (Bielza and Larranaga, 2014), deep learning (DL) (Bengio et al., 2013), decision trees (DTs) (Kotsiantis, 2013), clustering (Saxena et al., 2017), artificial neural networks (ANNs) (Dias et al., 2004), etc.

Each ML model is an intelligent computing mean that is trained to perform a well-defined task according to a set of observations. These intelligent models require a set of related data to extract knowledge about the problem at hand. The construction of these data is a crucial factor by which the performance of the model is judged. The more the data, the better the performance becomes.

All ML models undergo three principle steps: (1) receiving input data (signals), (2) processing these data, and finally (3) deriving outputs according to the handled task. To check if the system achieves a good learning level, an evaluation metric is computed. It is then tested on a number of patterns not previously observed and is then judged whether it has acquired a good generalization capability or not.

For any given application, there are a number of specific models that can perform better than the others. The choice of the best model for a well-determined task does not obey to any rule. Rather, there are only instructions on how these models proceed. Thus, there is no way to understand how to choose the best model for a problem.

While classic ML focuses on developing new models and techniques without regard to the resulting increase in complexity, automatic ML (AML), affirms that these tools can be employed in an easier manner. AML platforms computerize the majority of ML tasks in less time and implementation costs. Therefore, automatic ML has become a hot topic not only for industrial users, but also for academic purposes.

Fine-tuning or optimization is a key component to provide suitable models Hutter et al. (2019). AML framework addresses issues, such as the best ML model for different problems, model tuning or hyper-parameters optimization, etc. (Yao et al., 2019). Simple classical methods, Bayesian optimization and metaheuristics are among the most used tools of optimization in AML.

**Figure 1:**
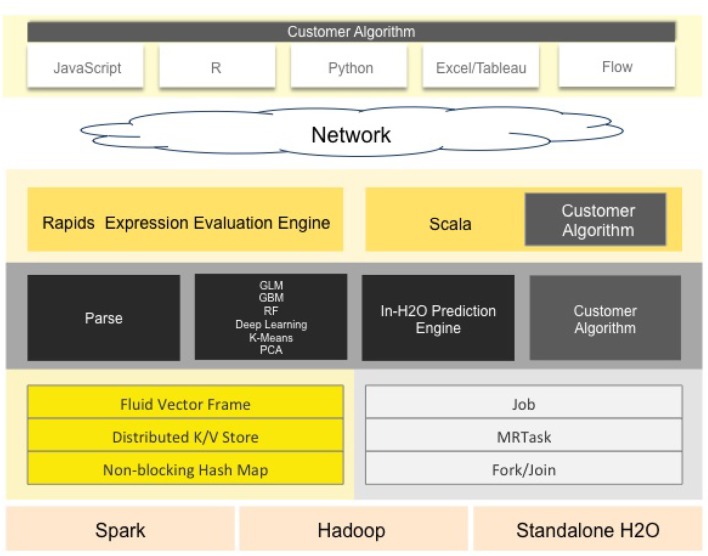
H2O's standard architecture. H2O is designed mainly on Java language with some blocks based on Python, JavaScript, R, and Flow. The software stack is composed of the top and bottom sections, divided by the network cloud. The top part highlights some of REST API customers, while the bottom illustrates the constituents undergoing the Java virtual machine (image courtesy of H2O.ai).

**Figure 2:**
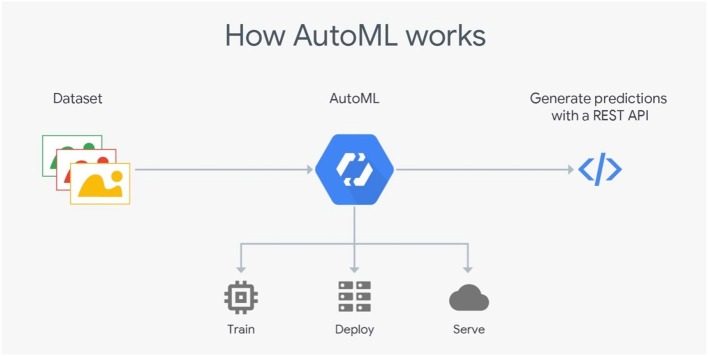
Google Cloud AutoML's standard architecture. Cloud AML offers a simple interface for inexperienced users to exploit models according to their needs. Using DNNs and genetic algorithms, Cloud AutoML trains machine learning models, deploys models based on user data, and stores trained data in cloud storage. The framework generates predictions with a REST API (image courtesy of Google Cloud).

**Figure 3:**
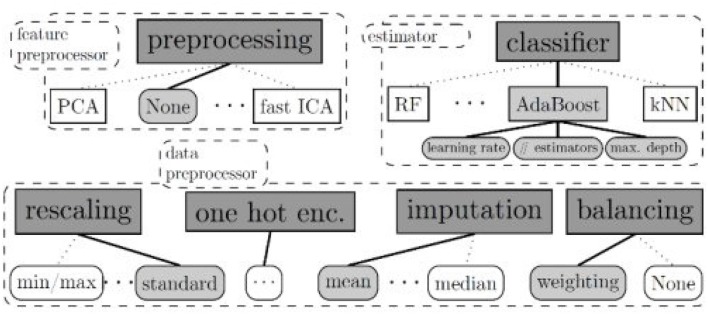
Auto-sklearns's standard architecture. Auto-sklearn employs Bayesian fine-tuning for hyperparameter settings. The program utilizes 15 classification approaches, 14 pre-processing techniques, and four feature engineering methods.

To develop such automated frameworks, researchers have developed and proposed several solutions e.g., H2O, Google Cloud AutoML, and Auto-sklearn depicted in Figures 1–3, respectively. These frameworks have certainly solved several problems but are still far from the strategy behind the human brain. What can be noticed throughout the enumerated techniques is that developers are using sophisticated ML models without reasoning; hence, no explainable AI.

- **H2O**
  H2O (Landry, 2018) is an open source machine learning platform for the enterprise. The platform contains a module that employs a set of well-defined algorithms to form a pipeline. It provides a specific graphical interface to set the appropriate model, the stopping criteria and the training dataset.
  It supports several linear and complex ML models, such as Deep Neural Networks (DNN), gradient boosting machines, etc. It also supports the Cartesian and random grid searches optimization techniques. It is designed based mainly on Java developing language with some blocks on Python, Javascript, R and Flow. The standard H2O architecture is visualized in Figure 1 (Landry, 2018).
  The H2O software stack depicted in Figure 1 is composed of numerous components that can be divided into two parts (top and bottom). The top part highlights some of REST API customers, while the bottom part illustrates the constituents undergoing the Java virtual machine.
  In spite of its ease of use especially for ML beginners and non-specialists, H2O still suffers from a lack of background in data science. Another drawback concerns the huge amount of employed resources. In fact, failures during complex executions are very likely to occur.
- **Google's Cloud AutoML**
  Cloud AutoML (Vinson, 2018) presents a series of products permitting inexperienced users to exploit well-qualified models obeying their business queries. It employs sophisticated capabilities of Google, such as transfer learning. It provides users with a simple interface so that they are able to learn, assess, improve, and unfold techniques according to their data. The products offered by this framework include AutoML Vision and video-intelligence, AutoML natural language and translation and AutoML Tables, etc. The standard Cloud AutoML's architecture is visualized in Figure 2 (Vinson, 2018).
  This framework is mainly based on deep neural networks (DNN) and genetic algorithms. It also asks users to respect a limit of training data size. For AutoML, tables data size should not surpass 100 Go.
- **Auto-sklearn**
  Auto-sklearn, proposed by Feurer et al. (2015), employs Bayesian fine-tuning for hyperparameter settings. It is an improved version of the scikit-learn system (a preceding automatic ML). The standard Auto-sklearn's architecture is visualized in Figure 3.
  There are 15 classification approaches, 14 pre-processing techniques and four feature engineering methods. Although its structure is advanced, this toolkit's package does not support natural language inputs. Therefore, it can not distinguish categorical data from digital data (Feurer et al., 2015).

Although the majority of preexisting ML frameworks have efficiently solved several problems, such as object recognition and image understanding, they are still far from simulating human brain processes. ML has attempted to mimic the brain as a model for computation, for instance neural networks algorithms, however ML is still not able to perform as well as the human brain. We propose a novel automatic ML framework called “BrainOS.” The proposed system architecture and operation is biologically inspired by neuron cells, designed at a very low level of abstraction.

## 3. BrainOS: A Novel Automatic ML Framework

Attracted by the strength of the human brain's ability to reason and analyze objects and ideas, we propose a novel automatic ML framework called “BrainOS.” The system's architecture and operation is inspired by the behavior of neuronal cells.

Since existing ML models have many challenges related to over-sized task-dependent training data and uninterpretable results, BrainOS addresses these shortcomings. Indeed, it provides a multidisciplinary approach able to deal with natural language processing (NLP) so that the gap between statistical NLP and many other disciplines necessary for understanding human language is minimized. Linguistics, commonsense reasoning, and affective computing are essential to analyze the human language. BrainOS involves symbolic as well as sub-symbolic techniques by employing models like semantic networks and conceptual dependency representations to encode meaning. Furthermore, it uses DNNs to deduce syntactic aspects from data.

### 3.1. High-Level BrainOS Model

Thanks to its anthropomorphic and data-adaptive power, BrainOS can be of great use in various types of applications, because it has the capability to react differently according to the user's profile and preferences. Data adaptation signifies the ability to pick out the most adequate mathematical model in terms of the received input data.

**Figure 4:**
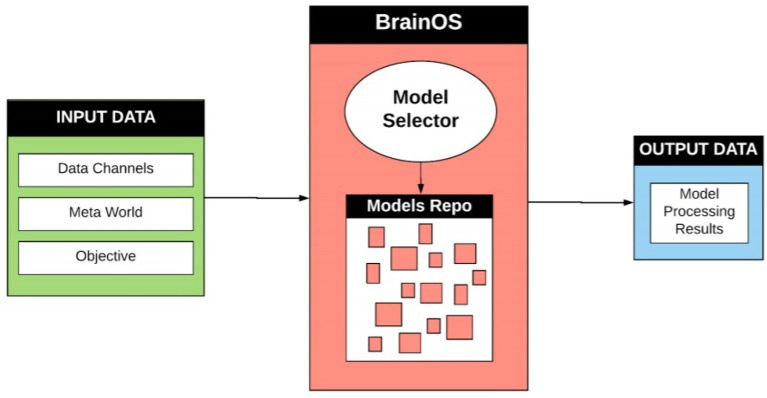
High-level Brain OS architecture. Input data information is received from various mixed input data channels. Real world context is retrieved from the meta-world container. The objective presents the aim of the processing problem and the desired outputs. The most appropriate model is then created and stored in the model repository for future use or chosen from a preexisting model within the repository. The output data contains the results and findings achieved after undergoing data processing.

The high-level BrainOS architecture is presented in Figure 4. The Input Data Layer is composed of data points coming from various source channels (sensors, videos, images, etc). When fed through this layer, the data undergo numerous stages of data retrieval and handling. For example, input points can be identified, typified, and pre-processed. Sampling techniques can also be employed at this level. The Data Processing Layer identifies a number of intelligent approaches according to the following stages:

- *Critic-Selector Mechanism*: combines input data types, processes history and objectives, researches knowledge and situational context to determine the most appropriate ML model for existing data and how the system should manage the processing resources.
- *Data handling using ML pipelines*: A series of vertical and horizontal pipelines to spread out the data can help prepare the data more quickly and efficiently.
- *Model training and/or transfer learning*: Not isolating algorithms and utilizing knowledge from a previous task to solve related ones increases efficiency and accuracy.

The Output Data Layer contains the results and the findings achieved after undergoing the Data Processing Layer.

BrainOS is adaptive to various data channels. It employs several data processing techniques and model selector components. Similar to the human brain, BrainOS uses an archive of data, knowledge and ML models. BrainOS is boosted by a complex qualifier-orchestrator meta-component. The critic-model selector is located within the orchestrator to give an answer to the question “What is the best tool to chose for a given problem?”.

Based on the human brain, which uses different neuronal areas to process input data, depending on the receptor type, the proposed infrastructure is founded on an ensemble of resources that are managed by the critic-selector (turned on and off), much in the manner the biological mind operates.

### 3.2. BrainOS Fundamental Architecture

**Figure 5:**
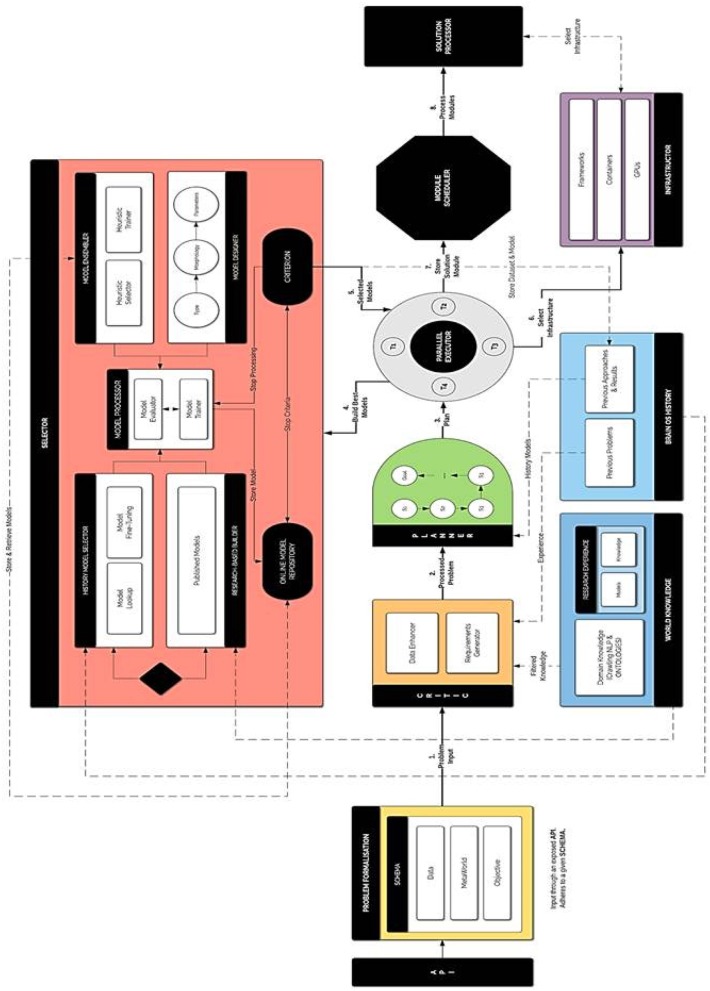
Detailed BrainOS architecture.

The key concept of BrainOS is its adaptability to the problem at hand. It selects the appropriate models for the nature of the input data. Figure 5 visualizes a more thorough overview about the architecture of the whole infrastructure. As shown in Figure 5, BrainOS topology is characterized by a number of components. In the next section, every component is detailed.

### 3.3. Problem Formalization Component

Problem formalization is the principle entry point of the system. It houses three sub-components: data, meta-world information, and task objective. These three components contain all the necessary related information associated with the data and the task to be processed. The input data is held within the data container while general and real world context data is held in the meta-world container. The task objective represents the primary aim of the problem to be processed and the desired outputs.

**Figure 6:**
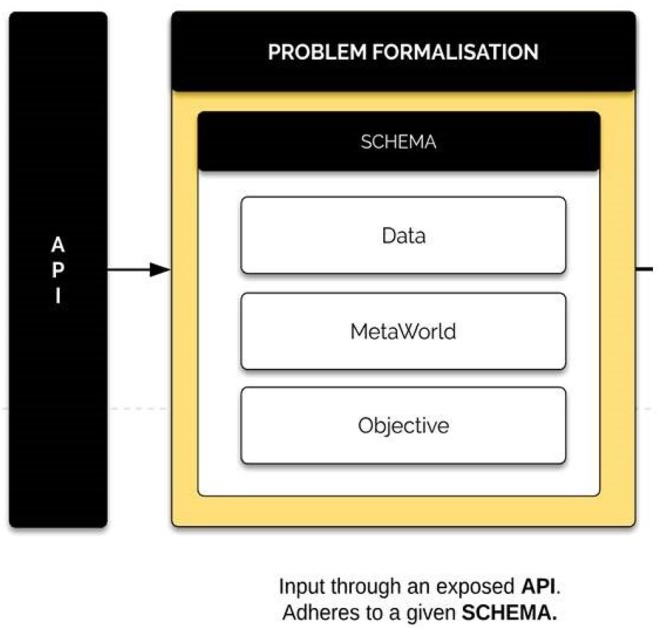
Problem formalization component. The problem formalization component includes mixed input data, general real-world data context contained within the meta-world container, and the major objective of the processing problem, as well as the desired outcomes.

For consistency, the input data points should comply to a specific schema. This can be done using an API to connect BrainOS to other ML packages to maintain the task's integrity and consistency. Figure 6 presents an example of the problem formalization component.

### 3.4. The Critic Component

**Figure 7:**
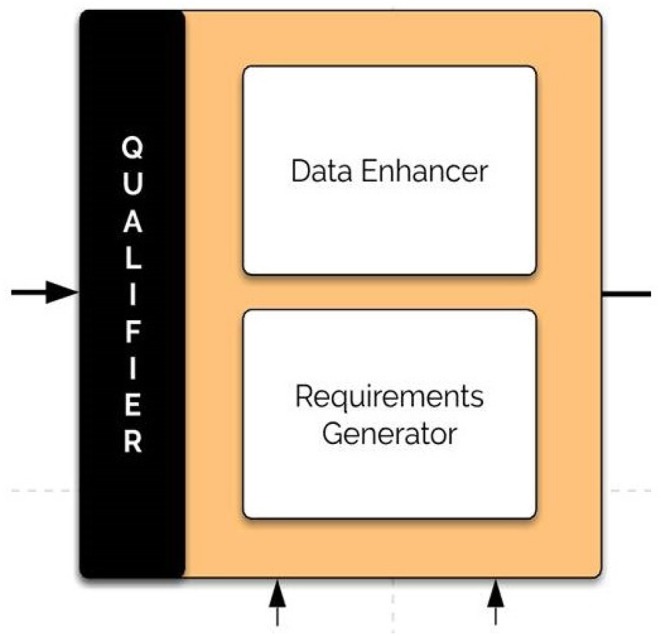
The qualifier (critic) component. The qualifier component enhances the datasets fed to the system and applies qualifications, imposes constraints, and builds requirements to achieve an intermediate.

The critic (qualifier) component utilizes the problem formulation and the BrainOS history (meta-world knowledge) to enhance the dataset fed to the system. It improves the data with antedate datasets, which complement the current input features in a module called the data enhancer. Furthermore, it applies qualifications, imposes constraints and builds requirements to achieve an intermediate. Figure 7 shows the architecture of the critic component.

### 3.5. History Database

**Figure 8:**
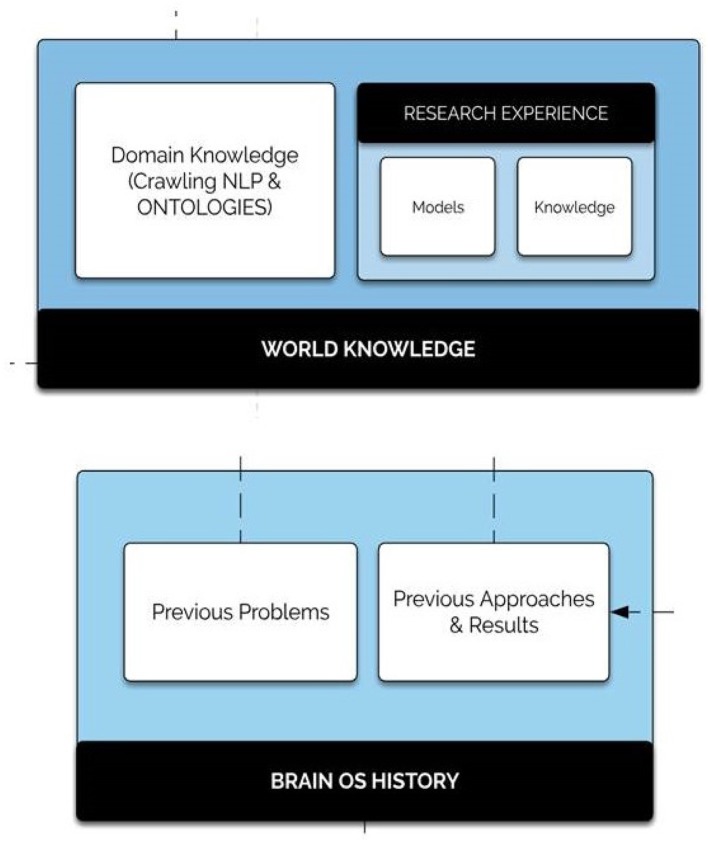
History database component. The history database component is comprised of world knowledge as well as the Brain OS history. The world knowledge sub-component contains the domain knowledge package of crawling NLP and ontologies as well as research experience comprised of stored models and more abstract research knowledge.

Proposing an adaptive learning system in a non-static space looks like the human's reasoning aspect. In fact, humans exploit their knowledge and experiences to find solutions to any kind of problem. Inspired by this extraordinary capability, BrainOS blends at least two memory sub-components: world knowledge and history. Figure 8 shows the architecture of the history database component.

1. *The BrainOS history:* includes the experience acquired over the system life cycle in terms of encountered data sets, previously employed models and achieved outcomes. Such a quick memory access resource is of great value especially in situations where the platform encounters problems already resolved. In this case, the system uses a “reflex response.”
2. *The world knowledge:* holds the “common sense” world knowledge, overlaying from general to domain-specific concepts. The domain knowledge package contains numerous fields within which the infrastructure requires a knowledge expert. The integrated research experience is comprised of models and inferences drawn from real world knowledge encompassing the following two components:
  - **Stored models**: include non-constrained previously discovered resources.
  - **More abstract research knowledge**: a big information field. It can be carried out on specific problem formulations, distinct problem solutions, or precise datasets.

### 3.6. The Planner Component

The Planner is based essentially on the processed problem and the history of used models. It is able to set the most adequate processing flow for the tackled problem according to the world knowledge, objective, and the similarity of the present task with those treated in the past.

As an example, for a problem of intent extraction from an image, the planner might prescribe the following steps:

- Run captioning algorithms on the image to obtain a narrativization of the image.
- Run object detection and activity recognition on the image.
- Run an algorithm to obtain an ontology for the previously extracted concepts.
- Infer intent using all the previously obtained entities and ontologies.

**Figure 9:**
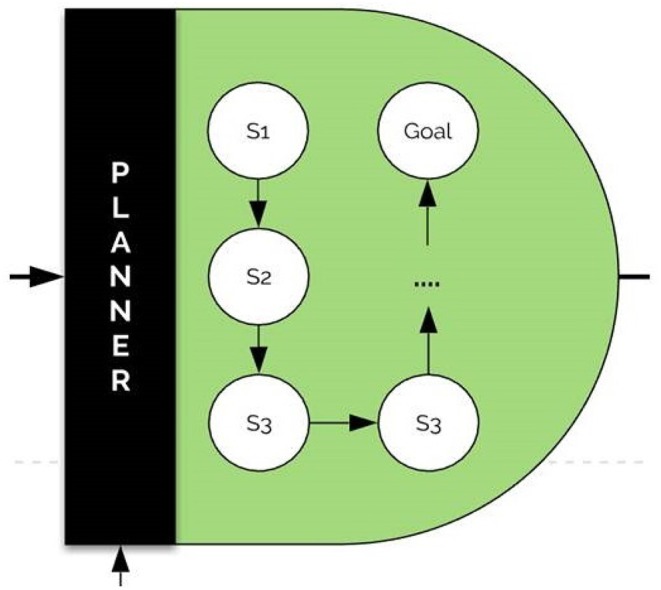
The planner architecture. The planner sets the most adequate processing flow for the problem according to world knowledge. Within the planner, special heuristic search algorithms can be run for the detection of the proper node sequences for a given task.

The planner plays the role of large bidirectional graph knowledge within which special heuristic search algorithms can be run for the detection of the proper node sequences for a given task. The architecture of the planner is visualized in Figure 9.

### 3.7. The Parallel Executor

The parallel executor plays the role of task scheduler. This component builds models, stores solution modules, and selects infrastructure. It manages when, what and how threads will be executed once they come from the selector.

**Figure 10:**
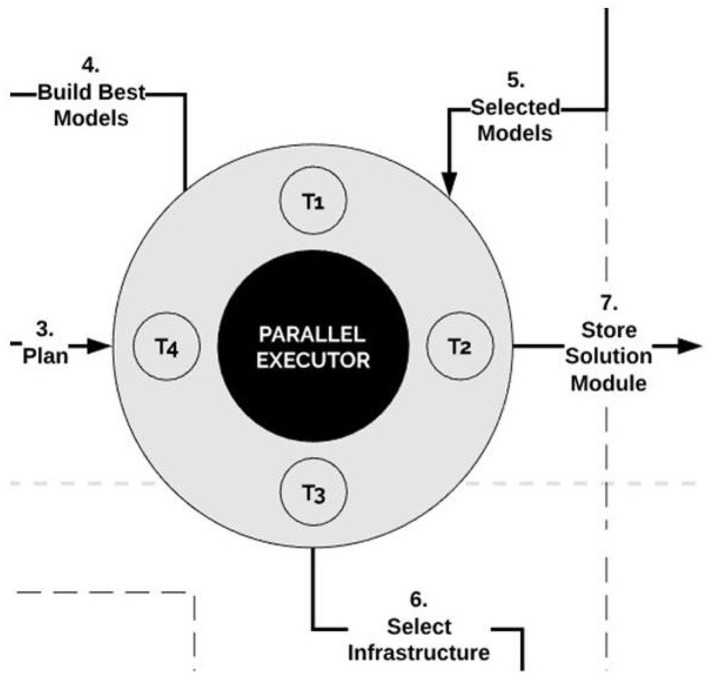
The parallel executor. The parallel executor creates new models or combines existing ones. It partitions corresponding tasks in parallel threads processing simultaneously.

The parallel executor triggers a number of threads for convenient structures. Based on the models provided by the selector, the executor creates new models or combines existing ones. It partitions the corresponding tasks in parallel threads processing simultaneously. The architecture of the parallel executor is visualized in Figure 10.

### 3.8. The Module Scheduler

The module scheduler receives threads sent by the parallel executor and plans a schedule for the solution's execution. This gives the ability of parallel execution using different resources.

### 3.9. The Selector Component

The Selector, the key component of BrainOS, picks out the adequate model according to the Problem Formulation. With the intention to provide suitable models, the Selector proceeds with the following steps in parallel:

1. Searching for an adequate model in BrainOS history. If a good fit is found, then the corresponding tool is optimized, trained, and evaluated.
2. Else, searching in the Research Knowledge including published papers and source codes. If a suitable candidate is found, then it is tuned, learned, and evaluated.
3. Building a tool from scratch after type and topology are defined. Thereafter, the model is tuned, trained, and assessed.
4. Performing an ensemble learning by combining several models which may give better findings than a higher accuracy model.

**Figure 11:**
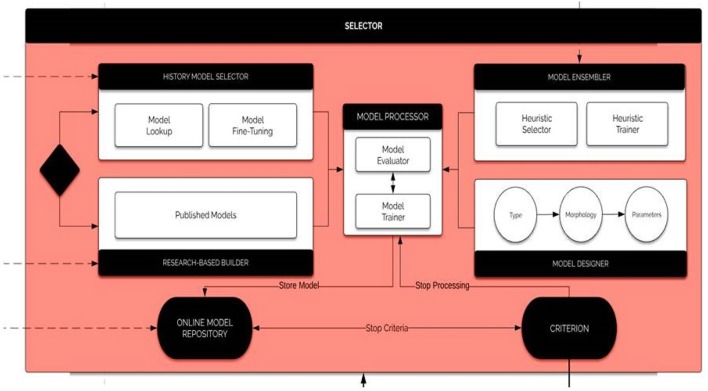
The selector. The selector runs the history model selector, the researched-based builder, the model ensembler, and the model designer in parallel. The history model selector searches for an adequate model in BrainOS history. The Research-Based Builder searches published papers and source code to find a suitable model. The model ensembler combines several models, which may give better findings than a higher accuracy model. The model designer builds tools from scratch. The model processor evaluates and trains the selected models.

Therefore, before the Selector adopts the solution model for the given Problem Formulation, it analyses whether there is a combination of models that can outperform the selected model. If the Selector finds such a model combination, then the model solution is an ensemble of models. The architecture of the module selector is visualized in Figure 11.

The selected ensemble of models, the problem formulation and the given precision are then archived in the BrainOS history. The four approaches are executed in parallel where every module records the best model within the online model repository.

The criterion determines whether the retrieval is a fitted enough approach according to the predetermined objectives, or when one of the modules should be excluded from the search. For each part of BrainOS processing plan, appropriate models are selected. It is advisable to furnish different specialized Domain Specific Instances of the selector, each one optimized for a specific domain knowledge or problem context. For instance, for classification purposes, SVM, K-means clustering, ANNs and other tools can be employed. For time-dependant problems, recurrent architectures, such as recurrent neural networks (RNNs) (Chouikhi et al., 2017) are highly recommended. To deal with feature engineering problems, independent component analysis (ICA) (Henriquez and Kristjanpoller, 2019), independent component analysis (PCA) (Kacha et al., 2020), autoencoders (AEs) (Xu et al., 2016), matrix factorization, and various forms of clustering.

Concerning optimization tasks, there are many useful techniques, such as evolutionary computation (Chouikhi et al., 2016), global optimization, naive optimization, etc.

### 3.10. The Orchestrator Component

From a high level of abstraction, the BrainOS plays the role of an orchestrator-centered infrastructure as it monitors overall models. It is arranged in a graph to pick out the processing paths. The proposed framework seems to be powerful as it can employ any approach from supervised to unsupervised learning, reinforcement learning, search algorithms, or any combination of those.

The orchestrator is a meta-component which merges input data, processes history and objectives, and researches knowledge and situational context to determine the most appropriate ML model for a given problem formulation. The orchestrator is comprised of four components: models selector, problem qualifier, planner and parallel executor.

## 4. Interpretations

Our evaluation of BrainOS focuses on the following questions:

**Question 1** *Flexibility and adaptability*: Is BrainOS capable enough to deal with a large variety of application areas?

**Question 2** *Fast convergence*: When dealing with a certain task, does BrainOS proceed quickly or it takes much time to converge?

**Question 3** *Accuracy*: How does BrainOS ensure the achievement of accurate results?

### 4.1. Flexibility and Adaptability

One of the most important characteristics of the BrainOS is its flexibility to handle several issues. BrainOS can be adapted for a large array of existing problems, and also extended for new approaches. Here, we provide just a small subset of possible application areas for the BrainOS. It can be applied to Anthropomorphism in Human/Machine Interaction problems including personality emulation and emotional intelligence. Moreover, BrainOS is relevant in dealing with brain disease diagnostics and treatment (e.g., Alzheimer, Parkinson Disease, etc.), automated manufacturing systems, energy management, etc.

In fact, the inner memory modules, incubated within the BrainOS architecture, store previous experiences and knowledge. This gives our platform the possibility to solve any kind of application, even those with a high-level of abstraction. What specifies the proposed paradigm over the state of the art, is the consistency with conceptual data, such as NLP. Indeed, it addresses the shortcomings of the existing models in solving many contextual tasks. Additionally, it provides a plenty of ML models, each of which performs in a specific field.

### 4.2. Fast Convergence

BrainOS can decrease the execution time. If a problem was previously tackled and another problem in the same context is about to feed to BrainOS, the model previously employed can be directly found in the BrainOS history and used to solve the new task. In this case, there is no need to proceed to the selector and the subsequent components. Furthermore, one of the common challenges of automatic ML systems is to quickly decide how to choose the model that best fits the given task. BrainOS encompasses a selector component which automatically and directly chooses better models according to the task at hand. This can be gainful in terms of run time. Furthermore, BrainOS supports parallel execution by launching several threads simultaneously through the parallel executor component. This can save much time and hasten data processing.

### 4.3. Accuracy

BrainOS holds many components, which constitute levels through which the data circulates. At the majority of these levels, there is a storage of historical processing and models and knowledge from world experience. Recording previous models and their findings gives a priori indications about what model to use. Furthermore, BrainOS provides several optimization techniques as well as ML models capable of affording high generalization capability. It is also possible to carry out an ensemble learning by executing many models at the same time and taking the best one.

### 4.4. Availability and Scalability

Data Processing Service is responsible for collecting data from different input channels, decompressing it, and storing it for later usage. There is a large number of data channels which can send data to the BrainOS. Thus, on the Cloud, there is a need for high scalability in recording this data, and there will also be a demand to store a large amount of it. There are different technologies which can support this, but the most suitable ones that can enable the constant increase of inputs and high parallelism of incoming data are those based on the Publish/Subscribe Paradigm. In this specific case of data processing, the inputs will act as data publishers while the BrainOS which processes the data, as a subscriber.

## 5. Empirical Results

Currently the implementation of AML models, such as Google's AI solution is likely to be susceptible to high latency, computational cost and power consumption. This is due to the huge data flow presented by larger data sets. The big issue, which the industry will not overcome easily, is that it is using digital arithmetic units and Boolean gates, which themselves are a mismatch with how neurons and synapses work. This represents, therefore, a poor approach to implementing deep neural architectures. To continue solving more complex problems, using increasingly more hardware is mandatory yet unsustainable. The proposed BrainOS is under the way of implementation. We are designing and testing some BrainOS modules, and we will gather all the modules into one framework. For example, we are working with a completely new architecture for Deep Neural Networks (DNN), which we call Deep Cognitive Neural Network (DCNN) (Howard et al., 2019).

### 5.1. Deep Cognitive Neural Network (DCNN)

**Figure 12:**
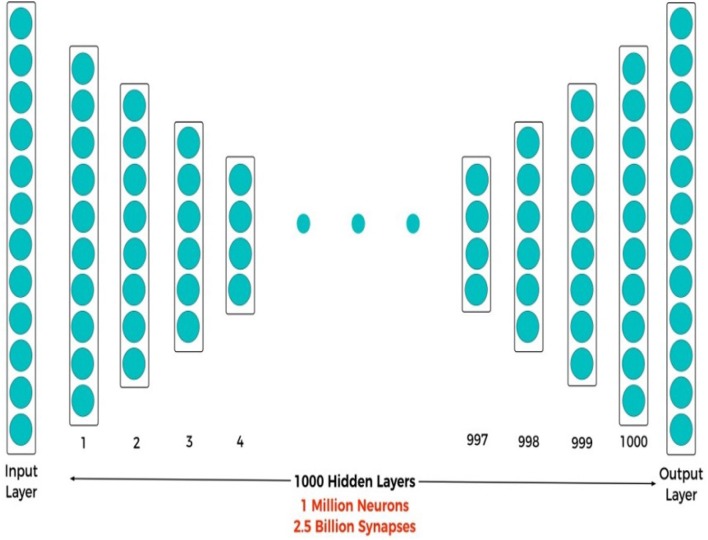
DCNN architecture (1,000 hidden layers, 1 million neurons, and 2.5 billion synapses).

DCNN is one of the new ML models exhibiting characteristics similar to the human brain, such as perception and reasoning and is a much better fit for building Neural Networks. The value of this new architecture is that big data analysis can be run near real-time on small devices, such as mobile phones and IoT devices. The proposed DCNN architecture, shown in Figure 12, is comprised of one million neurons and 2.5 billion synapses. DCNN has a remarkable property of concurrently acquiring highly energy-efficient implementation, fast decision-making, and excellent generalization (long-term learning). DCNN is highly energy-efficient in computing with ultra-low energy requirements that can easily be implemented in both hardware and software, as its neurons can be represented by simple equations consisting of addition, subtraction, and division operations. A highly energy-efficient implementation of shallow neural networks using complementary metal-oxide semiconductor (CMOS) or Probabilistic CMOS (PCMOS) technology has revealed that they are up to 300× times more efficient in terms of energy performance product (EPP). The substantial gain per-operation is proportional, which depends on the entire application, where large gains are expected with deep structures for large scale processing.

### 5.2. DCNN Fast Decision-Making

**Figure 13:**
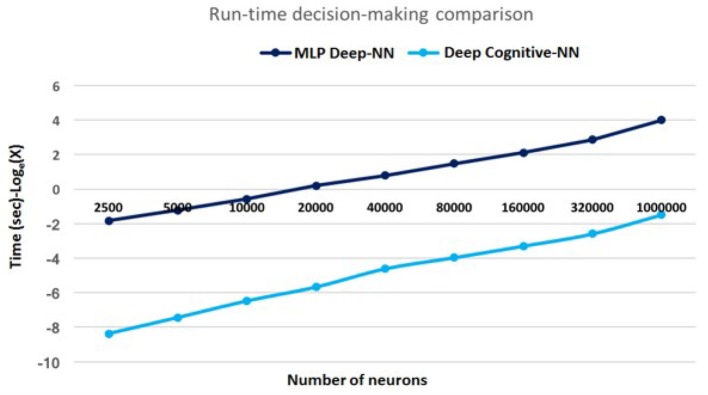
Decision making speed: for very large scale DNN processing, simulation results of DCNN has shown 300× faster decision-making as compared to the state-of-the-art Multi-Layer Perceptron (MLP) based deep neural network comprising one million neurons and 2.5 billion synapses.

DCNN was trained and tested using the state-of-the-art MNIST dataset (LeCun et al., 1998). The decision making results are depicted in Figure 13. It can be seen that for very large scale processing, DCNN has shown up to 300× faster decision-making as compared to the state-of-the-art Multi-Layer Perceptron (MLP) based deep neural network.

### 5.3. DCNN Integration With the Reasoning Algorithm

**Figure 14:**
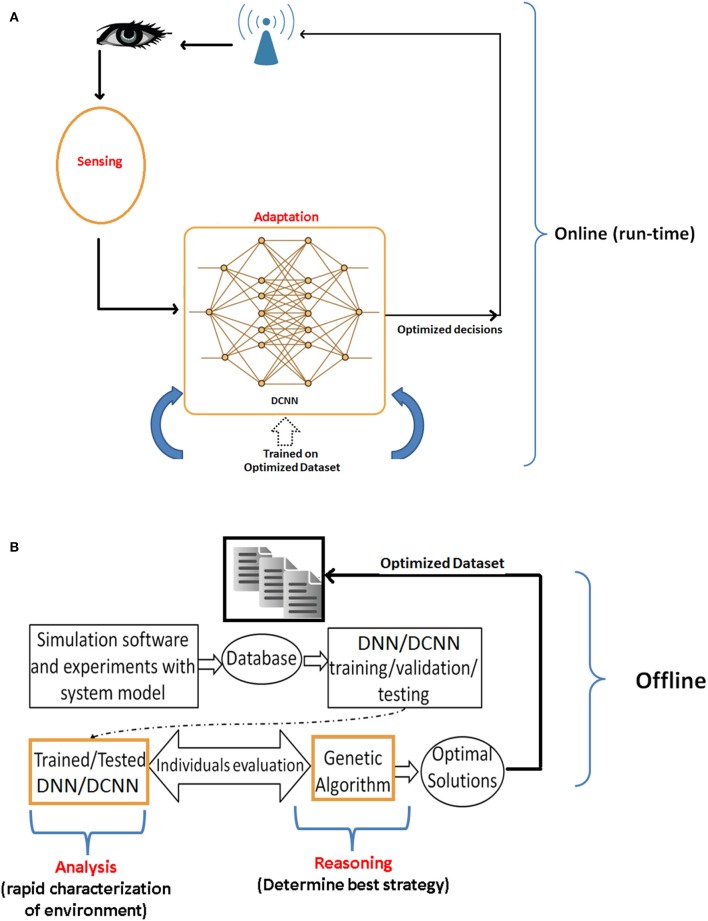
DCNN based optimized decision-making. **(A)** DCNN based real-time optimal adaptation. **(B)** Optimized dataset extraction: the data is first collected for learning which helped GA based reasoning process to build optimized dataset.

Another unique property of the developed DCNN is its quick adaptability and convergence behavior when integrated with reasoning algorithms to acquire human-like computing (both perception and reasoning simultaneously) in real-time. Large scale simulation reported up to 80× faster decision-making. The simulated reasoning/optimization framework is demonstrated in Figure 14. Figure 14A shows the DCNN based sensing and adaptation procedure, trained on an optimized dataset produced by the optimization framework. The optimization framework is shown in Figure 14B, which is responsible for analysis and reasoning. In this framework, the learning module assists the reasoning process in deciding the best configurations to be used in new upcoming situation. Whereas, the reasoning module [e.g., genetic algorithm (GA)] uses learning module to maximize the utility function. The proposed framework is used for an optimized and autonomous power control in wireless uplink systems. Simulation results demonstrated significant performance improvement of DCNN + GA framework as compared to DNN+GA, in terms of real-time decision making. Specifically, in an offline optimization mode, DCNN took 0.28 s/decision as compared to DNN's 2 min/decision. Nevertheless, once the DCNN is trained on an optimized dataset, it performed 300× time faster than DNN as shown in Figure 14. More details on the optimization framework and dataset are comprehensively presented in Adeel et al. (2016).

We believe that our proposed DCNN is an optimal choice for future ultra-low power and energy efficient devices capable of handling massive arrays of mathematical calculations in real-time for both generalized learning and optimization applications. To acquire more flexibility for dealing with a variety of applications, we are currently implementing the DCNN regression model along with the designing and testing of other BrainOS modules. Lately, we will gather all the modules in one framework.

## 6. Conclusion

Our work was motivated both by the intellectual goal of creating a model of human intelligence that better resembles how the brain and cognition works as well as the related practical goal of building a more effective machine learning approach; an automatic-ML approach in particular. While ML and AI approaches have generally been premised on duplicating brain and cognitive functions, their varied suitability for different kinds of problems means that no one model is adequate for all problems. The way forward as many have supposed long ago, is to figure out how to select an approach (which might be one or a system of models), in an automatic, rational/explainable manner, for any particular problem at hand, to elicit optimal solutions to that problem. This means the selection and calibration (i.e., parameter selection) of a system/architecture of models. The BrainOS system described in this paper differs from existing automatic ML tools in what it automates and how it does so. It proceeds from existing taxonomies of approaches in the automatic ML literature, to develop its own architecture. Preliminary studies have convinced us that BrainOS can deal with complex high-level problems, such as natural language processing.

## Author Contributions

NH contributed to the design of the proposed approach. NC was responsible for the state-of-the-art review and the paper write-up. AA conceived and co-developed the original idea of DCNN and DCNN based optimized decision-making. KD contributed substantially to the writing and revising of the manuscript. AHo co-designed the proposed architectural model. AHu was responsible for the overall planning and direction of the proposed approach, including the DCNN framework.

### Conflict of Interest

The authors declare that the research was conducted in the absence of any commercial or financial relationships that could be construed as a potential conflict of interest.
